# Acute coronary syndrome during active infusion of andexanet alfa

**DOI:** 10.1097/01.JAA.0000000000000354

**Published:** 2026-06-23

**Authors:** Vishal Dalal, Gwang-Yee J. Hu, Rocco A. Panico, Michael B. Rodricks

**Affiliations:** At Robert Wood Johnson University Hospital Somerset in Somerville, NJ, **Vishal Dalal, Rocco A. Panico**, and **Michael B. Rodricks** practice critical care medicine, and **Gwang-Yee J. Hu** is a critical care pharmacist. **V. Dalal** is also an adjunct instructor in the Rutgers School of Health Professions Department of Physician Assistant Studies and Practice in Piscataway, NJ, and is a clinical instructor in the Department of Medicine at Rutgers Robert Wood Johnson Medical School in New Brunswick, NJ. **M.B. Rodricks** is an associate professor in the Division of Acute Care Surgery at Rutgers Robert Wood Johnson Medical School. **G.Y. J. Hu** is a clinical assistant professor at the Ernest Mario School of Pharmacy in Piscataway, NJ. V. Dalal discloses service as a consultant for Teleflex Medical Incorporated. The authors have disclosed no other potential conflicts of interest, financial or otherwise.

**Keywords:** acute coronary syndrome, andexanet alfa, apixaban, factor Xa, STEMI, thrombosis

## Abstract

Now withdrawn from the US market, andexanet alfa was initially approved by the FDA in 2018 to reverse life-threatening bleeds associated with factor Xa (FXa) inhibitors, specifically apixaban and rivaroxaban. Observed effects of andexanet alfa include a reduction in anti-FXa activity resulting in hemostatic efficacy. However, thrombotic events including myocardial infarction and deep venous thrombosis, typically occurring 6 to 30 days after infusion, have also been observed. This case presents the first known patient to develop an ST-segment elevation myocardial infarction while actively receiving infusion of andexanet alfa, which was an approved therapy at the time of the patient's treatment.

## CASE

An 80-year-old man presented to the ED with a 2-day history of dark tarry stools as well as one episode of hematemesis associated with syncope.

**Figure:**
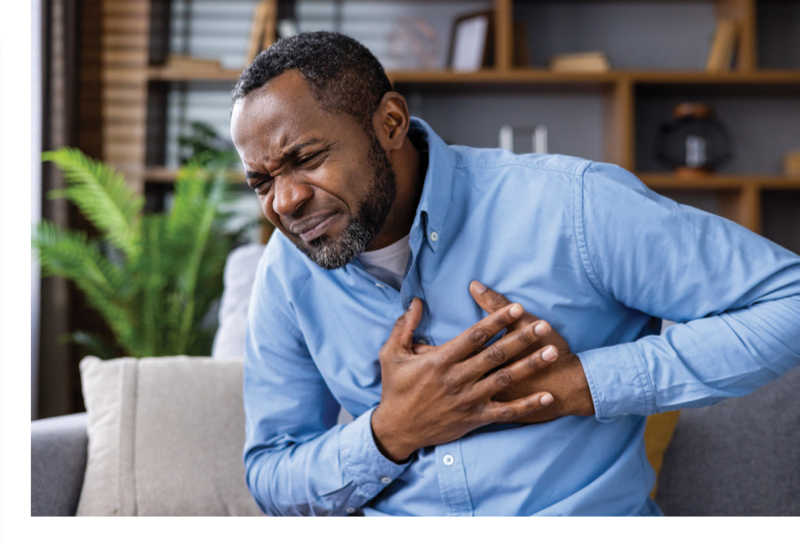
No caption available.

### History

The patient had a past medical history of class I obesity, hypertension, hypothyroidism, and chronic renal insufficiency. One week prior to presentation, the patient had been hospitalized for a provoked deep venous thrombosis (DVT) from immobility. He had been discharged home and compliant with apixaban 5 mg twice daily and aspirin 81 mg daily.

### Physical examination and diagnostic testing

**FIGURE 1.**
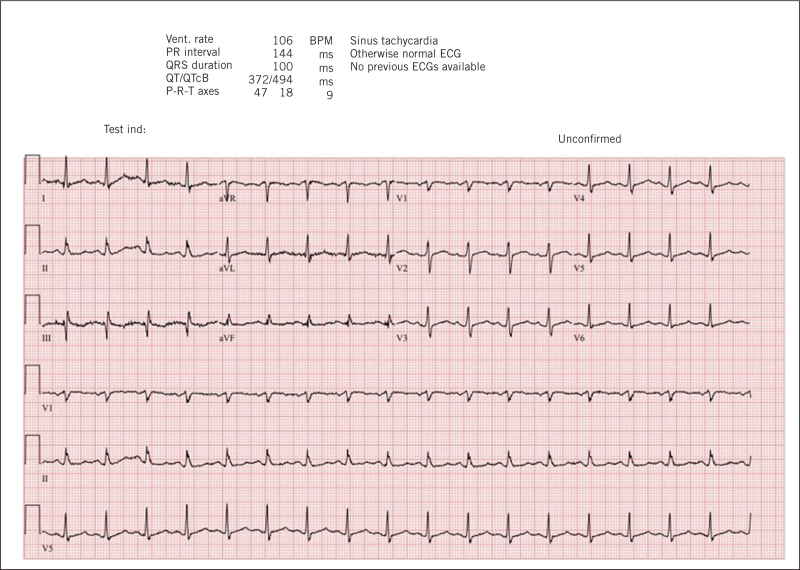
Initial 12-lead ECG at the time of the patient's presentation to the ED

Upon admission to the ED, the patient was tachycardic, with a heart rate of 114 beats/minute, and hypotensive, with a BP of 94/58 mm Hg. The patient was ill-appearing with a decreased level of consciousness. His rectal examination was hemoccult positive. His abdominal examination was benign. Pertinent laboratory values in the ED included a hemoglobin of 7.8 g/dL, decreased from 13.5 g/dL 5 days prior when the patient was hospitalized and diagnosed with DVT, and a creatinine of 1.7 mg/dL, which was similar to his baseline. An initial 12-lead electrocardiogram (ECG) was notable for sinus tachycardia and a prolonged QT interval. Additionally, the ECG showed a notched S wave in lead II and T wave flattening in the inferior leads, findings thought to be secondary to tachycardia and anemia (Figure 1).

### Management

The patient was given 1 L of normal saline, was transfused with 2 units of packed red blood cells, and immediately taken for an emergent esophagogastroduodenoscopy (EGD), which revealed three nonbleeding duodenal ulcers. Two hemostatic clips were placed on the largest of the three ulcers. The patient was felt to be at considerable risk of rebleeding given the location and nature of the ulcers. Due to the risk of rebleeding and concern for a uremic bleeding tendency associated with the patient's chronic renal insufficiency, he was treated with 0.3 mcg/kg of desmopressin upon admission to the ICU.

**Box 1:**
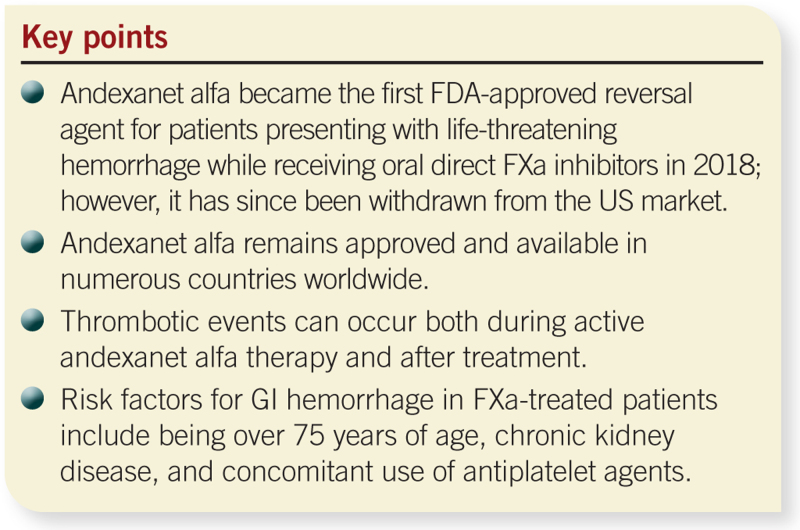
No caption available.

**FIGURE 2.**
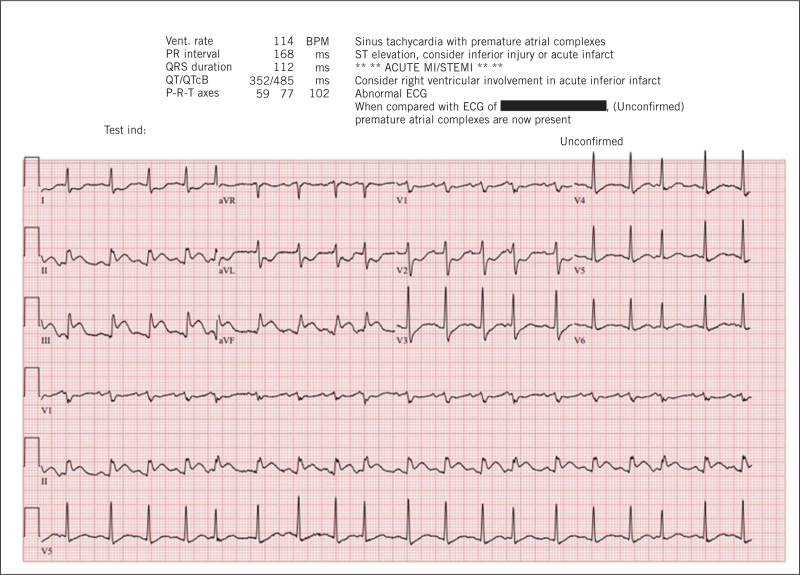
Repeat 12-lead ECG at the time of radiating mid-sternal chest pain and during infusion of andexanet alfa

Following the EGD, due to the patient's risk of rebleeding, labile hemodynamics, and recent (less than 12 hours) dose of apixaban, the patient was also administered 400 mg of andexanet alfa as a bolus followed by a 2-hour infusion. At the time this patient was treated, andexanet alfa was FDA-approved for this indication. Midway through the infusion he reported nausea and midsternal chest pain with radiation to his left scapula. A repeat 12-lead ECG revealed ST-segment elevation in lead II, III, and aVF (Figure 2). The andexanet alfa infusion was promptly discontinued, and arrangements were made for emergent cardiac catheterization. While awaiting catheterization, the patient continued to deteriorate with unstable ventricular arrhythmias requiring multiple defibrillations. Given his inability to protect his airway, he was intubated before coronary angiography.

The cardiac catheterization revealed 100% occlusion of the mid right coronary artery. The patient underwent angioplasty and placement of a single drug-eluting stent. Postprocedure, he was started on dual antiplatelet therapy with aspirin and clopidogrel. The patient was extubated on hospital day 2. Shortly after being extubated, the patient developed recurrent melena, anemia, and hypotension requiring vasopressors and further transfusion. He was taken emergently to interventional radiology, where a pseudoaneurysm of the gastroduodenal artery was identified and embolized. Over the subsequent week, the patient improved and was discharged home on hospital day 9. At discharge, he remained on dual antiplatelet therapy, and apixaban was not resumed.

## DISCUSSION

Oral direct FXa inhibitors were first approved by the FDA in 2011.^1^,^2^ FXa inhibitors do not require blood monitoring, have a lower risk of intracranial hemorrhage, and fewer drug interactions compared with warfarin. Given these benefits, the use of FXa inhibitors has increased since its approval.^3^ Despite lower rates of intracranial hemorrhage, FXa inhibitors have slightly higher rates of gastrointestinal (GI) hemorrhage depending on the agent and dose.^1^,^2^ Risk factors for GI bleeding in FXa-treated patients include being over 75 years of age, chronic kidney disease, and concomitant use of antiplatelet agents.^1^ The patient in this case had all of these risk factors.

Before the FDA approved andexanet alfa in 2018, 4-factor prothrombin complex concentrate (PCC) had been the reversal agent of choice in patients presenting with life-threatening hemorrhage. Although 4-factor PCC is the approved reversal agent for vitamin K antagonists in patients who present with life-threatening hemorrhage, it is not FDA approved for patients on FXa inhibitors.^4^ The 2018 FDA approval of andexanet alfa made it the first reversal agent for patients who present with life-threatening hemorrhage while being treated with oral direct FXa inhibitors; however, it has since been withdrawn from the US market, though it remains available in other countries. Andexanet alfa is a recombinant form of human FXa designed to rapidly bind FXa inhibitors and reduce anti-FXa activity.

The ANNEXA-4 trial was the first to demonstrate the efficacy of andexanet alfa in patients who presented with acute life-threatening hemorrhage while taking FXa inhibitors.^5^ This study included patients with any life-threatening hemorrhage, including GI bleeding. ANNEXA-4 demonstrated a decrease in anti-FXa activity with restoration of hemostasis.^5^ Reported complications included an increased risk of thrombotic events; 10% of patients had thrombotic complications, of which 2% were acute myocardial infarction.^5^ Similar rates of thromboembolic complications were noted in the ANNEXA-I trial.^6^ In this study, only patients with intracerebral hemorrhage were included. ANNEXA-I showed better control of hematoma expansion when compared with usual care (85.5% of whom received PCC).^6^ This study also showed that 10.3% of patients had adverse thrombotic events, including a 4.2% rate of myocardial infarction.^6^ Other thrombotic complications of andexanet alfa included ischemic stroke, DVT, and pulmonary embolism.^6^ In the usual care group, a 5.6% incidence of thrombosis was noted.^5^ We speculate that those in the andexanet alfa group had higher rates of thrombosis given the efficacy of the reversal agent. Because of significant rates of thrombotic complications, clinicians must carefully weigh the benefits against the risks of using this reversal agent in regions where it remains available. Reversal should generally be reserved for instances of life-threatening hemorrhage.

Although both the ANNEXA-4 and ANNEXA-I trials demonstrated thrombotic complications, patients most commonly developed thrombosis 6 to 30 days after treatment with andexanet alfa.^5^,^6^ The case presented here is unique due to the patient's ST-segment elevation myocardial infarction (STEMI) that occurred during the infusion of andexanet alfa, which may represent the first reported case of acute coronary syndrome (ACS) in a patient undergoing active therapy. This highlights the need for vigilance among clinicians in monitoring acute thrombotic events during active therapy.

While we acknowledge that there may have been confounders which contributed to the patient developing ACS during infusion, we suspect that andexanet alfa was a major contributor to the event. The patient received desmopressin to address platelet dysfunction given his chronic renal insufficiency and this may have predisposed the patient to a prothrombotic state. Because the patient had a 100% occlusion of the right coronary artery, and given that thrombi associated with occlusions of coronary vessels are typically fibrin rich, we suspect that the myocardial infarction was more closely related to the administration of andexanet alfa than desmopressin. We also recognize that the presence of acute anemia at the time of admission may have placed the patient at a higher risk for coronary ischemia. The presence of thrombosis on the coronary angiogram supports the hypothesis that the ACS resulted from an acute myocardial infarction rather than coronary ischemia alone.

## CONCLUSION

The use of FXa inhibitors has increased in recent years. Given the ubiquity of FXa inhibitors in clinical practice, the need to rapidly reverse their effects in the face of life-threatening hemorrhage will continue to grow. Although andexanet alfa has demonstrated a reduction in anti-FXa activity and been shown to achieve hemostatic efficacy, thrombotic events, including myocardial infarction, have also been described.^5^,^6^ Although most reported thrombotic events associated with andexanet alfa occur 6 or more days after therapy, this unique case reports an individual who experienced ACS during active infusion, highlighting the need for clinicians to monitor and remain vigilant for signs of thrombotic events both during active therapy and after treatment.

## References

[1] Patel MR Mahaffey KW Garg J Rivaroxaban versus warfarin in nonvalvular atrial fibrillation. *N Engl J Med*. 2011;365(10):883–891. 21830957 10.1056/NEJMoa1009638

[2] Granger CB Alexander JH McMurray JJV Apixaban versus warfarin in patients with atrial fibrillation. *N Engl J Med*. 2011;365(11):981–992. 21870978 10.1056/NEJMoa1107039

[3] Bruins Slot KM Berge E. Factor Xa inhibitors vs warfarin for preventing stroke and thromboembolism in patients with atrial fibrillation. *JAMA*. 2014;311(11):1150–1151. 24643605 10.1001/jama.2014.1403

[4] Goldstein JN Refaai MA Milling TJ Jr Four-factor prothrombin complex concentrate versus plasma for rapid vitamin K antagonist reversal in patients needing urgent surgical or invasive interventions: a phase 3b, open-label, non-inferiority, randomised trial. *Lancet*. 2015;385(9982):2077–2087. 25728933 10.1016/S0140-6736(14)61685-8 PMC6633921

[5] Connolly SJ Crowther M Eikelboom JW Full study report of andexanet alfa for bleeding associated with factor Xa inhibitors. *N Engl J Med*. 2019;380(14):1326–1335. 30730782 10.1056/NEJMoa1814051 PMC6699827

[6] Connolly SJ Sharma M Cohen AT Andexanet for factor Xa inhibitor-associated acute intracerebral hemorrhage. *N Engl J Med*. 2024;390(19):1745–1755. 38749032 10.1056/NEJMoa2313040

